# Clinical Outcomes and Patient-Reported Outcomes of Minimally Invasive Glaucoma Surgery Techniques Over the Past Decade

**DOI:** 10.7759/cureus.87872

**Published:** 2025-07-14

**Authors:** Poonam Singh, Bharti Sharma, Nilutpal Sarma, Deb Sanjay Nag, Abhishek Patnaik, Rashi Verma

**Affiliations:** 1 Ophthalmology, Tata Main Hospital, Jamshedpur, IND; 2 Anesthesiology, Tata Main Hospital, Jamshedpur, IND

**Keywords:** cataract, glaucoma, intraocular pressure, minimally invasive surgical procedures, patient-reported outcome measures

## Abstract

Minimally invasive glaucoma surgery (MIGS) has revolutionized glaucoma management over the past decade by offering safer, more efficient alternatives to traditional surgeries such as trabeculectomy. This review synthesizes clinical and patient-reported outcomes from 40 studies published between 2014 and 2025. MIGS techniques, including trabecular meshwork bypass stents (e.g., iStent, Hydrus), suprachoroidal shunts (e.g., CyPass), and subconjunctival devices (e.g., Xen), achieve intraocular pressure (IOP) reductions of 15-50%, reduce medication dependence by 0.4-1.8 drugs, and exhibit low complication rates (e.g., hyphema: ≤20%; hypotony: ≤15.4%). Combined MIGS-cataract procedures outperform standalone MIGS, with superior IOP control (additional 2-2.8 mmHg reduction) and lower reoperation rates (3% vs. 24% at two years). Patient-reported outcomes, though understudied, indicate enhanced quality of life, visual function, and ocular surface health. Challenges include variability in device efficacy and limited long-term data. Future research should prioritize standardized patient-reported metrics and diverse populations.

## Introduction and background

Glaucoma, a leading cause of irreversible blindness globally, affects over 80 million people, with intraocular pressure (IOP) reduction as the primary treatment goal [1]. Traditional surgeries such as trabeculectomy, while effective, carry significant risks, including bleb leaks, infections, and prolonged recovery. Minimally invasive glaucoma surgery (MIGS) emerged in the early 2010s to bridge this gap, offering micro-scale devices that enhance aqueous outflow with minimal tissue disruption. MIGS targets mild-to-moderate glaucoma patients, prioritizing safety, rapid recovery, and reduced medication burden [2].

Over the past decade, minimally invasive glaucoma surgery (MIGS) has seen exponential innovation, with devices now grouped by their mechanisms. Trabecular bypass stents such as the iStent (Glaukos Corporation, Laguna Hills, CA, USA) and Hydrus improve fluid drainage by enhancing conventional outflow through Schlemm’s canal. Suprachoroidal shunts, such as the CyPass, redirect fluid to the suprachoroidal space to reduce IOP. For subconjunctival drainage, subconjunctival filtration devices (e.g., the Xen Gel Stent) create new pathways in the subconjunctival space. Lastly, canaloplasty devices such as OMNI focus on catheterizing and dilating Schlemm’s canal to optimize fluid dynamics [3]. Together, these advancements mark a transformative era in glaucoma care, offering tailored, minimally invasive solutions.

This review evaluates clinical outcomes (IOP reduction, medication use, safety) and patient-reported outcomes (quality of life, visual function) across 40 studies (2014-2025). We assess device-specific efficacy, procedural contexts (standalone vs. combined with cataract surgery), and identify evidence gaps for future research. Standalone MIGS procedures appear to incur higher reoperation rates (up to 24% at two years) compared to combined procedures [4]. Medication use declined by approximately 0.4 to 1.8 fewer medications, with some series reporting medication-free rates between 22.6% and 80% [5]. Across a variety of device types, most notably iStent (including iStent inject) and Hydrus, most studies report maintenance or improvement of best-corrected visual acuity (BCVA), minimal vision-related complications, and generally transient adverse events (including hyphema, hypotony, and IOP spikes). Although less frequently assessed, patient-reported outcomes indicate improvements in quality of life, visual function, and ocular surface comfort when MIGS is combined with cataract surgery. This article provides an overview of the clinical outcomes and patient-reported outcomes of MIGS techniques over the past decade.

## Review

Literature search and screening

**Figure 1:**
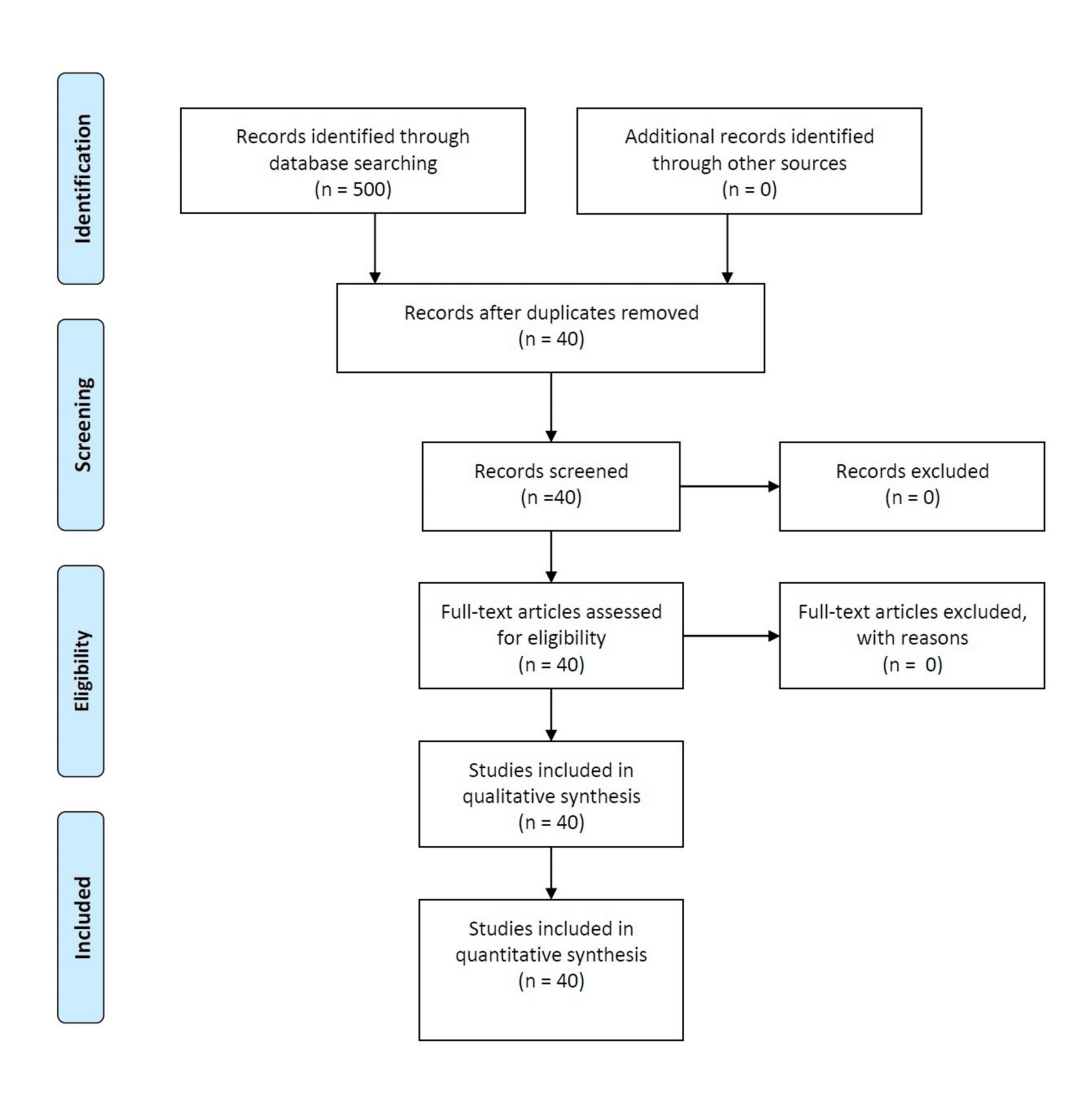
Preferred Reporting Items for Systematic reviews and Meta-Analyses (PRISMA) flow diagram.

A systematic search of the Semantic Scholar corpus, encompassing 126 million papers, was conducted using the query: “Clinical outcomes and patient-reported outcomes of MIGS techniques over the past decade.” This search yielded 500 potentially relevant publications. To ensure relevance and quality, the following strict inclusion criteria were applied: (1) studies involving adults (≥18 years) with any type of glaucoma; (2) interventions limited to MIGS alone or in combination with cataract surgery, excluding traditional glaucoma surgeries; (3) outcomes including clinical measures (e.g., IOP reduction, medication use), safety (e.g., complications), or patient-reported outcomes (e.g., quality of life); and (4) study designs restricted to randomized controlled trials (RCTs), cohort studies, systematic reviews, or case series with at least 10 participants. Following a holistic screening process, 40 studies met these criteria and were included for further analysis, as summarized in Table 1 [6-44]. The process of inclusion and exclusion is detailed in the Preferred Reporting Items for Systematic reviews and Meta-Analyses (PRISMA) flow diagram (Figure 1).

**Table 1:** Characteristics of the included studies. MIGS: minimally invasive glaucoma surgery; MIMS: minimally invasive micro-sclerostomy; OMNI: OMNI® Surgical System; GATT: gonioscopy-assisted transluminal trabeculotomy; iStent: iStent trabecular micro-bypass system (Glaukos Corp., Laguna Hills, CA); XEN: XEN gel stent (AbbVie Inc., Chicago, IL, USA); ICE2: iStent combined with phacoemulsification and endocyclophotocoagulation; PMS: PreserFlo MicroShunt

Study	Study design	MIGS device type	Patient population	Follow-up duration	Full text retrieved
Lee et al., 2017 [6]	Systematic literature review	iStent, iStent inject	Open-angle glaucoma	6–18 months (randomized controlled trials)	Yes
Ahmed et al., 2019 [7]	Randomized controlled trial	Hydrus, two iStents	Open-angle glaucoma	12 months	Yes
Pfeiffer et al., 2015 [8]	Randomized controlled trial	Hydrus	Open-angle glaucoma with cataract	24 months	Yes
Oo et al., 2024 [9]	Systematic review/meta-analysis	iStent, iStent inject, Hydrus, Kahook Dual Blade, Trabectome	Normal-tension glaucoma	6–36 months	Yes
Hu et al., 2022 [10]	Systematic review/network meta-analysis	Hydrus, iStent (first and second generation)	Open-angle glaucoma	End of follow-up (varied)	Yes
Reiss et al., 2019 [11]	Randomized controlled trial, prospective cohort	CyPass	Open-angle glaucoma with cataract	60 months	Yes
Höh et al., 2014 [12]	Prospective cohort	CyPass	Open-angle glaucoma with cataract	24 months	Yes
Neuhann et al., 2024 [13]	Retrospective review	iStent	Open-angle glaucoma, pseudoexfoliation glaucoma, ocular hypertension	10 years	Yes
Ahmed et al., 2022 [14]	Randomized controlled trial	Hydrus	Primary open-angle glaucoma with cataract	5 years	Yes
Melo Araújo et al., 2020 [15]	Randomized controlled trial	iStent inject	Primary open-angle glaucoma with cataract	24 months	Yes
Voskanyan et al., 2024 [16]	Prospective cohort	MIMS	Open-angle glaucoma, exfoliation glaucoma	52 weeks	Yes
Riss, 2022 [17]	Prospective cohort	MicroShunt	Primary open-angle glaucoma	2 years	Yes
Salimi et al., 2021 [18]	Prospective cohort	iStent, iStent inject	Primary angle-closure glaucoma with cataract	12 months	Yes
Cantor et al., 2023 [19]	Systematic review	iStent, OMNI, GATT, Kahook Dual Blade, Hydrus, Xen, PreserFlo, iTrack	Open-angle glaucoma	6–12 months (varied)	Yes
Gillmann et al., 2020 [20]	Systematic review/meta-analysis	Multiple MIGS	Open-angle glaucoma	Varied	Yes
Bicket et al., 2021 [21]	Systematic review	iStent, Hydrus, Trabectome, CyPass	Open-angle glaucoma	Short, medium, long-term	Yes
Lavia et al., 2017 [22]	Systematic review/meta-analysis	Multiple MIGS	Primary open-angle glaucoma, pseudoexfoliation, pigmentary glaucoma	12 months	Yes
Nichani et al., 2020 [23]	Systematic review	iStent, Hydrus	Mild-to-moderate open-angle glaucoma	1–2+ years	Yes
Aref et al., 2022 [24]	Systematic review	iStent, CyPass, Hydrus	Open-angle glaucoma with cataract	24 months	Yes
Qidwai et al., 2022 [25]	Retrospective review	ICE2, PMS, XEN-45	Primary open-angle glaucoma, secondary open-angle glaucoma, normal-tension glaucoma, ocular hypertension, primary angle-closure glaucoma	24 months	Yes
Jones et al., 2023 [26]	Retrospective observational	iStent inject, ICE2	Open-angle glaucoma	4 months	Yes
Le et al., 2019 [27]	Retrospective review	iStent, Hydrus	Open-angle glaucoma with cataract	24 months	Yes
Yang et al., 2022 [28]	Retrospective cohort		Glaucoma	2 years	Yes
Malvankar-Mehta et al., 2015 [29]	Systematic review/meta-analysis	iStent	Open-angle glaucoma with cataract	Varied	Yes
Turner et al., 2022 [30]	Retrospective review	iStent, XEN, Hydrus	Glaucoma with cataract	12–18 months	Yes
Oberfeld et al., 2024 [31]	Retrospective review	iStent, Kahook Dual Blade, Hydrus, combined MIGS	Severe glaucoma with cataract	12 months	Yes
Le and Saheb, 2014 [32]	Systematic review	iStent	Open-angle glaucoma with cataract	Varied	Yes
Buffault et al., 2019 [33]	Systematic review	XEN	Open-angle glaucoma, pseudoexfoliation glaucoma, pigmentary glaucoma	12 months	Yes
Al-Mugheiry et al., 2017 [34]	Prospective cohort	Hydrus	Open-angle glaucoma with cataract	16.8 months	Yes
Gołaszewska et al., 2021 [35]	Systematic review	Canaloplasty, iStent	Primary open-angle glaucoma	12–36 months	Yes
Bartelt-Hofer et al., 2020 [36]	Disease model/systematic review	Trabecular micro-bypass stent, IS	Primary open-angle glaucoma with cataract	1 year (model)	Yes
Richter et al., 2023 [37]	Systematic review	Trabecular MIGS	Open-angle glaucoma with cataract	2 years	Yes
Voykov et al., 2025 [38]	Systematic review	Kahook Dual Blade, iStent inject, Hydrus Microshunt	Glaucoma	Not specified in the abstract	Yes
Al Habash et al., 2020 [39]	Cross-sectional	Kahook Dual Blade, iStent, iStent inject, GATT	Glaucoma with cataract	Not specified in the abstract	Yes
Kazerounian et al., 2020 [40]	Retrospective cohort	Ab interno canaloplasty	Open-angle glaucoma (with or without cataract)	2 years	Yes
Mbagwu et al., 2024 [41]	Retrospective review	OMNI, Hydrus, iStent inject	Glaucoma with cataract	24 months	Yes
Chang et al., 2021 [42]	Retrospective review	Endoscopic cyclophotocoagulation, iStent, Kahook Dual Blade, Trabectome	Normal-tension glaucoma with cataract	2.5 years	Yes
Khaimi et al., 2017 [43]	Retrospective review	Canaloplasty	Open-angle glaucoma (with or without cataract)	3 years	Yes
Mosaed, 2017 [44]	Randomized controlled trial	CyPass	Mild-to-moderate glaucoma with cataract	2 years	Yes

Data Extraction

Data from the 40 included studies were extracted using a large language model to ensure efficiency and accuracy. The extracted information encompassed: (1) study design, setting, and participant demographics; (2) types of MIGS devices used and procedural details; (3) quantitative clinical outcomes, such as IOP reduction, medication reduction, and success rates; (4) frequency and severity of complications; and (5) patient-reported outcomes, including metrics related to quality of life and visual function. This structured approach facilitated a comprehensive synthesis of the evidence, allowing for comparisons across studies and devices.

Results

Study Characteristics

The 40 studies exhibited diverse designs, including 17 systematic reviews, six RCTs, six prospective cohort studies, 11 retrospective studies, and one cross-sectional study. The most frequently studied MIGS devices were the iStent trabecular micro-bypass system (18 studies), Hydrus (15 studies), iStent inject (eight studies), Kahook Dual Blade (six studies), and CyPass (five studies). Populations primarily consisted of patients with open-angle glaucoma (20 studies), with 14 studies involving MIGS combined with cataract surgery and three focusing on normal-tension glaucoma. Follow-up durations varied, with seven studies reporting 12-month outcomes, eight studies spanning 13-24 months, and seven studies extending beyond 24 months, up to 10 years.

Clinical Outcomes: IOP Reduction

MIGS procedures demonstrated significant IOP reductions, ranging from 0.1 to 30.2 mmHg, corresponding to 4-56% reductions from baseline. Greater reductions were observed in patients with higher baseline IOP, such as those in the minimally invasive micro-sclerostomy study, which reported a mean reduction of 10.5 mmHg [16]. Success rates, defined as ≥20% IOP reduction or IOP ≤18 mmHg, ranged from 45% to 96%, with Hydrus outperforming single iStent implants (p < 0.001) [7]. Combined MIGS-cataract surgery yielded 2-2.8 mmHg greater IOP reductions compared to cataract surgery alone [8,15]. However, standalone MIGS procedures were associated with higher reoperation rates, reaching 24% at two years [4]. The findings are summarized in Table 2.

**Table 2:** Effects on intraocular pressure reduction. MIGS: minimally invasive glaucoma surgery; MIMS: minimally invasive micro-sclerostomy; OMNI: OMNI® Surgical System; iStent: iStent trabecular micro-bypass system (Glaukos Corp., Laguna Hills, CA, USA); XEN: XEN gel stent (AbbVie Inc., Chicago, IL, USA); ICE2: iStent combined with phacoemulsification and endocyclophotocoagulation; PMS: PreserFlo MicroShunt

Study	MIGS device	Baseline intraocular pressure	Mean intraocular pressure reduction	Success rate
Lee et al., 2017 [6]	iStent, iStent inject	No mention found	No mention found	Relative risk = 1.38 (95% confidence interval = 1.18–1.63) for drop-free at 6–18 months
Ahmed et al., 2019 [7]	Hydrus, two iStents	23–39 mmHg	No mention found	Hydrus showed higher surgical success (p < 0.001)
Pfeiffer et al., 2015 [8]	Hydrus	No mention found	2.3 mmHg lower at 24 months versus cataract surgery (p = 0.0093)	80%: 20% or more intraocular pressure reduction at 24 months
Oo et al., 2024 [9]	Multiple	No mention found	2.1–2.44 mmHg at 6–36 months	No mention found
Hu et al., 2022 [10]	Hydrus, two iStents	No mention found	Hydrus: 2.21 mmHg; two iStents: 1.88 mmHg	No significant difference in medication-free status
Reiss et al., 2019 [11]	CyPass	No mention found	No mention found	46% 20% intraocular pressure reduction at 60 months
Höh et al., 2014 [12]	CyPass	21/<21 mmHg	-37% (uncontrolled), 0% (controlled) at 24 months	No mention found
Neuhann et al., 2024 [13]	iStent	18.6 ± 4.4 mmHg	12.9–19.0% at 10 years	77.8% intraocular pressure 18 mmHg at 10 years
Ahmed et al., 2022 [14]	Hydrus	No mention found	16.8 ± 3.1 mmHg at 5 years	49.5% intraocular pressure 18 mmHg without medications
Melo Araújo et al., 2020 [15]	iStent inject	~25 mmHg	7.0 mmHg (microstent), 5.4 mmHg (control) at 24 months	75.8% 20% intraocular pressure reduction (microstent)
Voskanyan et al., 2024 [16]	MIMS	27.9 ± 3.7 mmHg	10.5 mmHg (38%) at 52 weeks	82.1% qualified, 70.5% complete success
Riss, 2022 [17]	MicroShunt	25.7 ± 6.1 mmHg	9.9 mmHg at 1 year, 9.2 mmHg at 2 years	80.3% (1 year), 75.4% (2 years)
Salimi et al.,2021 [18]	iStent, iStent inject	18.8/18.7 mmHg	21%/25% at 12 months	45%/64% at 12 months
Cantor et al., 2023 [19]	Multiple	No mention found	-31% to -13.7% (6 months), -39% to -11.4% (1 year)	No mention found
Gillmann et al., 2020 [20]	Multiple	No mention found	15.3–50% (device-dependent)	No mention found
Bicket et al., 2021 [21]	Hydrus, iStent, CyPass	No mention found	Hydrus: 2.0 mmHg greater at long-term	Relative risk 1.6 (Hydrus), 1.4 (iStent), 1.3 (CyPass)
Lavia et al., 2017 [22]	Multiple	No mention found	3.4–4.1 mmHg (device-dependent)	No mention found
Nichani et al., 2020 [23]	iStent, Hydrus	No mention found	8–12 mmHg post-surgery	No mention found
Aref et al., 2022 [24]	Multiple	No mention found	20% unmedicated intraocular pressure lowering	No mention found
Qidwai et al., 2022 [25]	ICE2, PMS, XEN-45	18.5–20.5 mmHg	4.5–8.2 mmHg at 24 months	No mention found
Jones et al., 2023 [26]	iStent inject, ICE2	18.0 mmHg	4.0 mmHg at 4 months	No mention found
Le et al., 2019 [27]	iStent, Hydrus	No mention found	0.1–1.6 mmHg at 24 months	No mention found
Yang et al., 2022 [28]	Multiple	No mention found	Decreased in all groups	Reoperation: 3–24% at 2 years
Malvankar-Mehta et al., 2015 [29]	iStent	No mention found	4–27% (device-dependent)	No mention found
Turner et al., 2022 [30]	iStent, XEN, Hydrus	17.08 ± 4.23 mmHg	2.16 mmHg at 12–18 months	No mention found
Oberfeld et al., 2024 [31]	Multiple	16.7 ± 5.8 mmHg	3.2 mmHg at 12 months	47.5–87.5% (varied thresholds)
Le and Saheb, 2014 [32]	iStent	No mention found	No mention found	No mention found
Buffault et al., 2019 [33]	XEN	No mention found	25–56% (mean 42%) at 12 months	No mention found
Al-Mugheiry et al., 2017 [34]	Hydrus	18.1 ± 3.6 mmHg	2.8 mmHg at 16.8 months	80–96% (varied thresholds)
Gołaszewska et al., 2021 [35]	Canaloplasty, iStent	45.0 ± 12.1 mmHg	29.9–30.2 mmHg at 3 years	47.2–81% (varied thresholds)
Bartelt-Hofer et al., 2020 [36]	Multiple	No mention found	-2.05 to -4.85 mmHg at 1 year	No mention found
Richter et al., 2023 [37]	Trabecular MIGS	No mention found	1.6–2.3 mmHg at 2 years	No mention found
Voykov et al., 2025 [38]	Multiple	No mention found	1.8–1.9 mmHg (iStent inject, Hydrus)	No mention found
Al Habash et al., 2020 [39]	Multiple	No mention found	No mention found	No mention found
Kazerounian et al., 2020 [40]	Ab interno canaloplasty	20.24 ± 5.92 mmHg	6.57 mmHg at 2 years	80% off medication
Mbagwu et al., 2024 [41]	OMNI, Hydrus, iStent inject	No mention found	-4.96 to -6.64 mmHg at 24 months	No mention found
Chang et al., 2021 [42]	Multiple	13.7 mmHg	1.4 mmHg at 2.5 years	5.4–67.2% (criteria dependent)
Khaimi et al., 2017 [43]	Canaloplasty	19.7 mmHg	4.5–5.7 mmHg at 1–3 years	57.8–91.8% (varied thresholds)
Mosaed, 2017 [44]	CyPass	No mention found	7.4 mmHg at 2 years	No mention found

Clinical Outcomes: Medication Reduction

Medication burden decreased significantly post-MIGS, with reductions ranging from 0.4 to 1.8 fewer drugs. The highest medication-free rate (73%) was observed with Hydrus combined with cataract surgery [9]. Long-term data showed that iStent maintained a 33.3% medication-free rate at 10 years [13]. Multi-stent approaches, such as Hydrus or two iStents, consistently outperformed single-implant strategies in reducing medication use [7,10]. Table 3 summarizes the effect on the reduction in medications.

**Table 3:** Reduction in medications. MIGS: minimally invasive glaucoma surgery; MIMS: minimally invasive micro-sclerostomy; OMNI: OMNI® Surgical System; iStent: iStent trabecular micro-bypass system (Glaukos Corp., Laguna Hills, CA, USA); ICE2: iStent combined with phacoemulsification and endocyclophotocoagulation; PMS: PreserFlo MicroShunt

Study	MIGS device	Baseline medications	Medication reduction	Medication-free rate
Lee et al., 2017 [6]	iStent, iStent inject	No mention found	Mean difference = -0.42 (95% confidence interval = -0.60 to -0.23)	No mention found
Ahmed et al., 2019 [7]	Hydrus, two iStents	No mention found	-0.6 (Hydrus) at 12 months	22.6% more Hydrus subjects medication-free
Pfeiffer et al., 2015 [8]	Hydrus	No mention found	0.5 ± 1.0 (Hydrus + cataract surgery), 1.0 ± 1.0 (cataract surgery) at 24 months	No mention found
Oo et al., 2024 [9]	Multiple	No mention found	0.87–1.26 at 6–36 months	73% (Hydrus + cataract surgery), 38% (cataract surgery) at 24 months
Hu et al., 2022 [10]	Hydrus, two iStents	No mention found	No explicit quantitative value	No significant difference
Reiss et al., 2019 [11]	CyPass	No mention found	No mention found	No mention found
Höh et al., 2014 [12]	CyPass	No mention found	1.0–1.1 at 24 months	No mention found
Neuhann et al., 2024 [13]	iStent	1.83 ± 1.03	37.8–51.4% at 10 years	33.3% at 10 years
Ahmed et al., 2022 [14]	Hydrus	No mention found	0.5 ± 0.9 (Hydrus), 0.9 ± 0.9 (cataract surgery) at 5 years	No mention found
Melo Araújo et al., 2020 [15]	iStent inject	No mention found	-0.4 versus control at 24 months	66% (Hydrus), 46% (cataract surgery) at 5 years
Voskanyan et al., 2024 [16]	MIMS	1.8 ± 0.8	0.27 ± 0.7 at 52 weeks	No mention found
Riss, 2022 [17]	Micro Shunt	2.9 ± 1.1	0.6 ± 1.0 (1 year), 1.0 ± 1.3 (2 years)	No mention found
Salimi et al.,2021 [18]	iStent, iStent inject	No mention found	52%/50% at 12 months	No mention found
Cantor et al., 2023 [19]	Multiple	No mention found	No mention found	No mention found
Gillmann et al., 2020 [20]	Multiple	No mention found	No mention found	No mention found
Bicket et al., 2021 [21]	Multiple	No mention found	No mention found	No mention found
Lavia et al., 2017 [22]	iStent, Hydrus	No mention found	No mention found	No mention found
Aref et al., 2022 [24]	Multiple	No mention found	No mention found	No mention found
Qidwai et al., 2022 [25]	ICE2, PMS, XEN-45	2.0–2.9	0.5–2.0 at 24 months	No mention found
Jones et al., 2023 [26]	iStent inject, ICE2	1.8 ± 0.8	1.1 ± 0.9 at 4 months	No mention found
Le et al., 2019 [27]	iStent, Hydrus	2.1–2.6	0.3–1.1 at 6 months	No mention found
Yang et al., 2022 [28]	Multiple	No mention found	No mention found	No mention found
Malvankar-Mehta et al., 2015 [29]	iStent	No mention found	1.01–1.33	No mention found
Turner et al., 2022 [30]	iStent, XEN, Hydrus	2.68 ± 1.06	1.46 ± 1.32 at 12–18 months	No mention found
Oberfeld et al., 2024 [31]	Multiple	2.3 ± 1.9	1.8 ± 1.7 at 12 months	No mention found
Le and Saheb, 2014 [32]	iStent	No mention found	No mention found	No mention found
Buffault et al., 2019 [33]	XEN	No mention found	Reduction in all studies	No mention found
Al-Mugheiry et al., 2017 [34]	Hydrus	1.96 ± 0.96	0.04 ± 0.20 at 16.8 months	No mention found
Gołaszewska et al., 2021 [35]	Canaloplasty, iStent	No mention found	Significant reduction	No mention found
Bartelt-Hofer et al., 2020 [36]	Multiple	No mention found	No mention found	No mention found
Richter et al., 2023 [37]	Trabecular MIGS	No mention found	No mention found	No mention found
Voykov et al., 2025 [38]	Multiple	No mention found	No mention found	No mention found
Al Habash et al., 2020 [39]	Multiple	No mention found	Significant reduction (p < 0.001)	No mention found
Kazerounian et al., 2020 [40]	Mbagwu et al., 2024	Ab interno canaloplasty OMNI, Hydrus, iStent inject	1.92 ± 1.04	0.05 ± 0.23 at 2 years
Chang et al., 2021 [42]	Multiple	2	1.1 at 1.5 years	No mention found
Khaimi et al., 2017 [43]	Canaloplasty	2.1	0.4–0.6 at 1–3 years	No mention found
Mosaed, 2017 [44]	CyPass	No mention found	No mention found	No mention found

Safety Outcomes

Common complications included hyphema (≤20%), hypotony (8.8-15.4%), IOP spikes (≤32.7%), and stent obstruction (≤8.8%) [10,12,22]. Most complications were transient, with sight-threatening events, such as endophthalmitis, being rare (one case reported [26]). Surgical reoperations, often for stent malposition, were noted in nine studies [6,33]; however, overall, MIGS demonstrated a favorable safety profile compared to traditional glaucoma surgeries. The reported complications are presented in Table 4.

**Table 4:** Types of Complication

Study	Complications	Frequency	Severity	Required
Lee et al., 2017 [6]	Stent malposition/obstruction, intraocular pressure rise, hyphema, hypotony	No mention found	Transient, not vision-threatening	No mention found
Ahmed et al., 2019 [7]	Secondary glaucoma surgery, best-corrected visual acuity loss 2 lines	3.9% (two iStents), 2 eyes (Hydrus), 1 eye (two iStents)	No mention found	No mention found
Pfeiffer et al., 2015 [8]	Peripheral anterior synechiae, inflammation, Descemet membrane folds, iris erosion	Peripheral anterior synechiae: 9	Minor, transient	3 glaucoma surgeries for intraocular pressure
Oo et al., 2024 [9]	No mention found	–	–	–
Hu et al., 2022 [10]	Device malposition/obstruction, peripheral anterior synechiae, hyphema, uveitis, macular edema	Peripheral anterior synechiae: 15.3% (Hydrus), others <6%	Generally not sight-threatening	No mention found
Reiss et al., 2019 [11]	Sight-threatening events, best-corrected visual acuity loss, visual field mean deviation worsening	3 events (2 Micro-Stent, 1 control)	Serious, but few	No mention found
Höh et al., 2014 [12]	Hypotony, micro-stent obstruction	Hypotony: 15.4%, obstruction: 8.8%	Transient, not sight-threatening	11% required surgery
Neuhann et al., 2024 [13]	Secondary glaucoma surgeries, age-related macular degeneration, optic atrophy	9 surgeries, 5 age-related macular degeneration/atrophy	No sight-threatening/device-related	Surgery as needed
Ahmed et al., 2022 [14]	Endothelial cell loss, peripheral anterior synechiae, device malposition	No mention found	Peripheral anterior synechiae not affecting intraocular pressure	No mention found
Melo Araújo et al., 2020 [15]	No mention found	–	–	–
Voskanyan et al., 2024 [16]	Iris plugging, intraocular pressure spikes, others rare	Iris plugging: 18, intraocular pressure spikes: 15	Mild-to-moderate	Pilocarpine, laser, viscoelastic removal
Riss, 2022 [17]	Increased intraocular pressure, hyphema	No mention found	No mention found	4 reoperations
Salimi et al.,2021 [18]	No mention found	–	–	–
Cantor et al., 2023 [19]	No mention found	–	Most transient, non-serious	–
Gillmann et al., 2020 [20]	No mention found	–	–	–
Bicket et al., 2021 [21]	Vision loss (CyPass)	No mention found	–	–
Lavia et al., 2017 [22]	Intraocular pressure spikes	0–32.7%	Generally minimal	Additional surgery
Nichani et al., 2020 [23]	Stent obstruction, inflammation	No mention found	No mention found	No mention found
Aref et al., 2022 [24]	No mention found	–	–	–
Qidwai et al., 2022 [25]	Buttonhole, cystoid macular edema, inflammation, keratitis, branch retinal vein occlusion	No mention found	Transient	Nonsteroidal anti-inflammatory drugs, steroids
Jones et al., 2023 [26]	Endophthalmitis, hypotony, choroidal detachment	1 endophthalmitis	Transient	No mention found
Le et al., 2019 [27]	Bleeding, hyphema, stent repositioning, intraocular pressure spikes	Intraocular pressure spikes: 3.9–17%	Generally transient	Repositioning, trabeculectomy
Yang et al., 2022 [28]	No mention found	1–2%	No mention found	No mention found
Malvankar-Mehta et al., 2015 [29]	No mention found	–	–	–
Turner et al., 2022 [30]	No mention found	–	–	–
Oberfeld et al., 2024 [31]	No mention found	–	–	–
Le and Saheb, 2014 [32]	Stent obstruction/malposition	Infrequent	Transient	Observation, secondary procedures
Buffault et al., 2019 [33]	Hypotony, choroidal detachment, hyphema, bleb leak, malignant glaucoma	Hypotony: 3%, others <2%	Transient, some severe	Needling (32%), repeat surgery (5.7%)
Gołaszewska et al., 2021 [35]	Micro-hyphema, Descemet membrane detachment, intraocular pressure, stent issues	Micro-hyphema common, Descemet membrane detachment 3.3%	Generally transient	No mention found
Mbagwu et al., 2024 [41]	No mention found	–	–	–
Chang et al., 2021 [42]	Inflammation, hypotony, hyphema, edema, cystoid macular edema	See table	Transient	No mention found
Khaimi et al., 2017 [43]	Hyphema, cataract, intraocular pressure spikes, hypotony	No mention found	Low, no serious events	No mention found
Mosaed, 2017 [44]	No mention found	–	–	No vision-threatening events

Patient-Reported Outcomes

Patient-reported outcomes highlighted the benefits of MIGS beyond clinical metrics. BCVA was maintained or improved in 97.5% of cases, with BCVA loss being rare (1.2-2.5%) and unrelated to the devices [7,8,26]. Quality of life improved for 79% of patients post-MIGS, with significant gains in glaucoma-specific metrics, such as reduced photophobia and improved mobility, as reported by Jones et al. [26,39]. Reduced medication use correlated with improved ocular surface health, alleviating symptoms such as dryness and redness [26,39].

Comparative Device Performance

Hydrus and multi-stent devices demonstrated superior IOP reduction (2.21 mmHg) and medication-free rates (22.6-80%) compared to single iStent implants (1.88 mmHg) [7,10]. Standalone MIGS had higher reoperation rates (24% at two years [4]) compared to combined procedures (3% [14]). Long-term efficacy was notable with iStent combined with cataract surgery, achieving a 77.8% success rate at 10 years [13].

Discussion

Clinical Implications

MIGS has emerged as a significant advancement in glaucoma management, offering a less invasive approach compared to traditional surgeries while effectively lowering IOP and reducing the dependence on medications [1]. The combination of MIGS with cataract surgery demonstrates synergistic benefits, offering enhanced efficacy compared to either procedure alone, which optimizes the outcome for patients with both conditions [8,15]. Specific MIGS devices, such as Hydrus and multi-stent approaches, have shown superior IOP control, while others, such as XEN gel stent (AbbVie Inc., Chicago, IL, USA) (Xen), are more suitable for refractory cases [7,10]. The low complication profile of MIGS makes it a valuable option for a wide range of glaucoma patients. The reported improvement in quality of life indicates that MIGS can enhance patient comfort and overall well-being, contributing to a more positive patient experience [26,39].

Limitations

We acknowledge several limitations that affect the overall interpretation and generalizability of the findings. Heterogeneity in the definition of “success,” particularly concerning IOP thresholds, makes it challenging to compare outcomes across different studies, potentially skewing overall efficacy assessments [26,32,39]. The limited reporting of patient-reported outcomes constrains the evaluation of the full impact of MIGS on patients’ lives, with only a few studies addressing quality of life and visual function [26,32,39]. The presence of industry-funded trials introduces a potential bias, as such trials may be more likely to report favorable outcomes, potentially overstating the efficacy of specific devices [8,14]. There is a need for longer-term data, particularly for newer MIGS devices such as Hydrus and OMNI, to establish their durability and long-term effectiveness [18]. The limited studies exploring MIGS in specific glaucoma types, such as angle-closure glaucoma, underscore the need for more targeted research in diverse glaucoma populations [18].

Future Directions

To advance the field and address current limitations, several key areas for future research are highlighted. Standardizing patient-reported outcome metrics is crucial to better capture the holistic impact of MIGS on patients’ lives. Using validated tools such as the National Eye Institute Visual Functioning Questionnaire 25 would enable more consistent and comparable data across studies [18]. Gathering long-term data (more than five years) for newer devices such as Hydrus and OMNI® Surgical System (OMNI) is essential to understand their long-term efficacy and safety profiles [18]. Addressing the gap in knowledge regarding the efficacy of MIGS in diverse populations, such as those with angle-closure glaucoma, is needed to tailor treatment strategies and improve outcomes in these groups [18]. Future studies should focus on exploring the effectiveness of MIGS in various stages of glaucoma, including advanced cases, to better define the role of MIGS in the spectrum of glaucoma management.

## Conclusions

MIGS has established itself as a safe, effective, and patient-friendly option for managing glaucoma, particularly in those with mild-to-moderate disease and coexisting cataract. Clinical outcomes demonstrate meaningful IOP and medication reductions while maintaining visual acuity and minimizing serious complications. Combined procedures with cataract surgery offer the most favorable profiles in terms of efficacy and safety. Although data on patient-reported outcomes are still emerging, preliminary findings suggest improvements in visual function and quality of life. Further high-quality, long-term studies focusing on diverse populations and standardized quality of life metrics are warranted to better inform clinical practice and health policy.
